# From Colonial Research Spirit to Global Commitment: Bayer and African Sleeping Sickness in the Mirror of History

**DOI:** 10.3390/tropicalmed5010042

**Published:** 2020-03-10

**Authors:** Ulrich-Dietmar Madeja, Ulrike Schroeder

**Affiliations:** Bayer AG Pharmaceuticals, Müllerstrasse 178, 10785 Berlin, Germany; ulrike.schroeder@bayer.com

**Keywords:** African sleeping sickness, development of treatment, suramin, medical history, political history

## Abstract

In the early 20th century, a series of epidemics across equatorial Africa brought African sleeping sickness (human African trypanosomiasis, HAT) to the attention of the European colonial administrations. This disease presented an exciting challenge for microbiologists across Europe to study the disease, discover the pathogen and search for an effective treatment. In 1923, the first “remedy for tropical diseases”—Suramin—manufactured by Bayer AG came onto the market under the brand name “Germanin.” The development and life cycle of this product—which today is still the medicine of choice for Trypanosoma brucei (T.b), hodesiense infections—reflect medical progress as well as the successes and failures in fighting the disease in the context of historic political changes over the last 100 years.

By the middle of the 19th century, several international health conferences had already set themselves the goal of protecting the colonial countries from tropical diseases such as smallpox, cholera, plague and yellow fever. Then, at the beginning of the 20th century after several epidemics across equatorial Africa, the colonial powers became aware of an additional disease, African sleeping sickness (human African trypanosomiasis, HAT), which posed a threat not only to Europeans travelling to the colonies but also to the local population, as well as to the economic value of the colonies themselves for their ruling countries.

The colonial powers’ response was immediate for humanitarian reasons, but humanitarianism reflected the sentiment of the time—“a mixture of benevolent condescension and outright racism” [1] towards the local population—as illustrated by the colonialists’ claim that they were “saving hapless Africans from the diseases that plagued them” [1]. However, their intervention was also based on practicalities, such as the need to protect their source of manpower in the thinly populated equatorial zone. Transport at this time relied on human porters or canoes because pack animals were unable to survive in regions infested with tsetse flies. This new epidemic and the resulting deaths among the local population not only caused transport problems, but also aggravated agricultural development, thus threatening to thwart plans to further exploit the colonies [1,2]. However, the turn of the century was also the heyday of microbiology in Europe. Motivated scientists were eager to discover new pathogens, explore life cycles of vectors and develop vaccines and potential new treatments. African sleeping sickness, which posed such a problem for colonial administrations, thus presented a great challenge for microbiologic research [1].

In fact, the challenge was so great as well as worrying that from 1901 to 1913 the colonial administrations sent a total of 15 special research missions to Africa to study the new disease [3,4]. Eight of these missions were from Great Britain, who hosted a major conference on African sleeping sickness in London in 1907–1908 attended mainly by scientists from Britain, France, Germany and Portugal. Recent research findings as well as potential new drugs for treatment and prophylactic measures were discussed. At the same time, Germany and Great Britain were also active on a political level. They implemented steps to stop Africans infected with the disease from crossing borders and then, in 1911, they signed an agreement outlining their plans to jointly fight African sleeping sickness in West Africa.

Bayer, which had been involved in the production and sale of synthetic dyes since its founding in 1863, expanded its activities at the end of the 19th century to include other business areas. In 1891, the chemist and later CEO of Bayer, Carl Duisberg (1861–1935), established a scientific laboratory and efficient research department in Wuppertal-Elberfeld where dyestuffs and their intermediates were developed, as well as pharmaceuticals (e.g., acetylsalicylic acid, Aspirin™). Unlike the first discoveries of medicines which were based on success in the treatment of disease symptoms, research at this time increasingly focused on combating newly discovered pathogens. It was German physician and Nobel prize winner Paul Ehrlich (1854–1915) who defined the term chemotherapy as the “creation of a chemical that would attack a specific pathogen” [1]. He demonstrated that dyes could also be effective against specific pathogens. The so-called trypan dyes were identified as being effective against animal trypanosomes but turned out to be too toxic to be used for treatment of human African trypanosomiasis.

In 1909, Wilhelm Roehl (1881–1929), Ehrlich’s former assistant, asked Duisberg to help by providing dyes and money for animal experiments. Heinrich Hoerlein, head of Bayer’s Pharmaceutical Department since 1910, realized the importance of Roehl’s work and employed him. In 1916, with the help of a small team of chemists, he developed the first effective drug for the treatment of African sleeping sickness: the compound “Bayer 205,” a colorless and odorless urea derivative which was later named Suramin.

In 1921, the compound was successfully tested on animals and passed on to the Hamburg Clinic for Tropical Diseases for further testing. There, the English engineer Christopher G. James had been suffering from sleeping sickness for over eight months. James had contracted the infection in Rhodesia and seemed to have little chance of survival. After only a few injections with “Bayer 205,” however, Christopher G. James was well again and was able to travel back to Africa [5].

Encouraged by this success, Bayer sent an expedition to South Africa to carry out the necessary field trials on site, despite the difficult conditions after the First World War. The German microbiologist and pharmacologist Friedrich Karl Kleine (1869–1951), an expert in African sleeping sickness research, took the lead. In November 1921, the expedition, equipped with 30 kg of “Bayer 205,” set off from Cape Town to Rhodesia (Figure 1, Figure 2 and Figure 3).

The scientific experiments began in January 1922 and showed that oral applications only had a temporary effect and that injections were much more effective. The results were so convincing that the Governor General of Belgian Congo even invited the expedition to continue its work in the southern Congo region. In 1923, the “remedy for tropical diseases”—Suramin—came onto the market under the patriotic name “Germanin” (Figure 4).

After the First World War, scientific collaborations across Europe slowly resumed. As part of its war reparations and in an attempt to regain possession of its former colonies, Germany expressed its willingness to reveal the secret formula of Suramin. After consultations, France and Great Britain agreed not to accept the offer [1]. A French pharmacologist Ernest Fourneau (1872–1949) succeeded in reverse-engineering the drug based on patents that Bayer had taken out and renamed it “Fourneau 309” [1,6,7,8]. The pharmaceutical company Rhône-Poulenc marketed it then under the trade name “Moranyl.”

Despite being effective in the treatment of the first stage of African sleeping sickness, Suramin was not able to reverse the disease course once the trypanosomes had penetrated the blood-brain barrier. As such, Suramin remained the treatment of choice for acute cases of African sleeping sickness [4].

The availability of Suramin played a significant role in controlling the 1920s epidemic and subsequent outbreaks and in significantly decreasing the number of reported cases until the 1940s. At that time new treatments also became available. Pentamidine, discovered in 1940, started to be used for the treatment of the first stage of T.b. *gambiense* infections. In 1949, Melarsoprol was discovered and used for the treatment of both T.b. *gambiense* and T.b. *rhodesiense* infections. Due to being derived from arsenic, this treatment had many undesirable side effects, some even fatal. Furthermore, increased resistance to Melarsoprol had been observed in certain focal disease areas, particularly in central Africa.

Over the years, the colonial powers introduced extensive screening of populations at risk by mobile teams and implemented early vector control measures [9]. The disease was under control by the mid-1960s with fewer than 5000 cases reported across the African continent (Figure 5) [5].

By the mid-1960s, most countries affected by African sleeping sickness became independent. Support from the colonial powers ended and most African countries experienced an era of political instability and economic downturn. The effect on health services and on the control and prevention of endemic tropical diseases was disastrous. Consequently, disease control programs were stopped and population screening declined considerably. This situation provoked a new epidemic of African sleeping sickness.

During the 1950s to early 1970s, extensive insecticide spraying was the method of choice for vector control and resulted in a significant reduction in tsetse fly populations, as they are the vector for African sleeping sickness, but concerns about the environmental effect of DDT (Dichlordiphenyltrichlorethan) led to a worldwide ban in the late 1970s.

Since the mid-1970s, decreasing vector control and disease prevention as well as screening and treatment of populations at risk resulted in a steady increase in the number of reported cases of African sleeping sickness. This most recent epidemic lasted until the late 1990s. Around the year 2000, the scale of African sleeping sickness had once again almost reached the levels of the epidemics seen at the beginning of the 20th century (Figure 5) [11,13].

Suramin was added to the “WHO Model List of Essential Medicines” in 1979 and became the medicine of choice for T.b. *rhodesiense* infections, even though the published clinical evidence to support the use of the product remained limited. With no more disease control programs in place in Africa, the demand for medicines to treat African sleeping sickness decreased to a very low level. The only new product, Eflornithine, was registered in 1990. This molecule has shown to be less toxic than Melarsoprol but is only effective against T.b. *gambiense*. Furthermore, the treatment regimen is complex and difficult to apply.

In 1999, pharmaceutical companies started to question whether to continue manufacturing products which were in such low demand. Aventis proposed a substantial increase in price for further supplying Pentamidine, a second-line option to Suramin since 1937. The production of Melarsoprol, used for the treatment of second-stage disease caused by T.b. *rhodesiense* when the central nervous system is involved, became uncertain because of ongoing discussions in Europe over manufacturing the required raw materials containing arsenic. On several occasions, Bayer, which was still producing Suramin on the grounds that no alternative treatments were available, also threatened to halt production [14].

After the colonial-motivated push for research and control of African sleeping sickness in the early 20th century, the increased efforts of WHO, national control programs, bilateral cooperation and nongovernmental organizations (NGOs) have been able to reverse the curve of reported cases after the year 2000, ending the last major epidemic. The availability of novel diagnostic tools such as rapid diagnostic tests (RDTs) and raising awareness in the political will of local countries to fight African sleeping sickness played a crucial role in advancing disease control.

WHO also started public-private partnerships with pharmaceutical companies to secure the supply of the essential medicines to treat African sleeping sickness. In 2001, WHO and the pharmaceutical companies Bayer AG and Aventis (now Sanofi) reached an agreement to provide their medicines to treat African sleeping sickness free of charge for endemic countries. Pharmaceutical companies started to engage beyond donation of medicines and to provide much needed financial contributions for screening of focal disease areas by mobile intervention teals, surveillance and disease mapping, as well as training and public awareness programs. Within the period 2000–2018 the number of reported cases dropped by 95%.

The development of new treatment regimens and products has been part of this success story. In 2009, the new Nifurtimox Eflornithine Combination Therapy (NECT) was introduced for the treatment of T.b. *gambiense* infections. It simplified the use of eflornithine by reducing the duration of treatment and the number of intravenous infusions. In the same year, the combination of Nifurtimox (produced by Bayer) and originally registered for the treatment of American trypanosomiasis, with Eflornithine (produced by Sanofi) was included in the “WHO List of Essential Medicines” and is currently recommended as first-line treatment for the T.b. *gambiense* form. Since 2009, both pharmaceutical companies have been providing these products free of charge to WHO for endemic countries. The products are packed in a patient treatment kit containing all the material needed for their administration in remote rural areas [15].

In 2005, the development of Fexinidazole as the first-all oral treatment for T.b. *gambiense* infected patients started. On 10 July 2019, the WHO added the product to the “WHO List of Essential Medicines.” Fexinidazole is indicated for first stage and non-severe second stage and it can simplify and facilitate case management of human African trypanosomiasis caused by T.b. *gambiense* [16].

Under the lead of WHO and the implementation of efficient disease control programs, the number of reported cases of African sleeping sickness dropped below 10,000 (9878) in 2009 for the first time in 50 years and continued to decline with only 997 new cases reported in 2018, the lowest level since the start of systematic global data-collection 80 years ago [15].

One hundred years ago, Suramin was discovered as the first effective treatment of African sleeping sickness. The history of this product and the motivation for fighting African sleeping sickness have changed significantly over time and reflect the historic medical and political changes. In 2012, the “WHO Roadmap on Neglected Tropical Diseases (NTDs)” set the goal to achieve the sustainable elimination of African sleeping sickness as a public health problem by 2020. This goal was reached by WHO and partners a concerted high-impact approach.

New clinical developments are essential to overcome the development of cross-resistance of currently used substances and to advance treatment options. Surely, one of these “will finally put suramin to rest and ease this esteemed great-grandfather of chemotherapy into a well-deserved retirement” [17]. Along with other interventions, the next goal of achieving disruption of transmission of African sleeping sickness, as outlined in the “WHO NTD Roadmap 2030,” seems now to be very feasible.

## Figures and Tables

**Figure 1:**
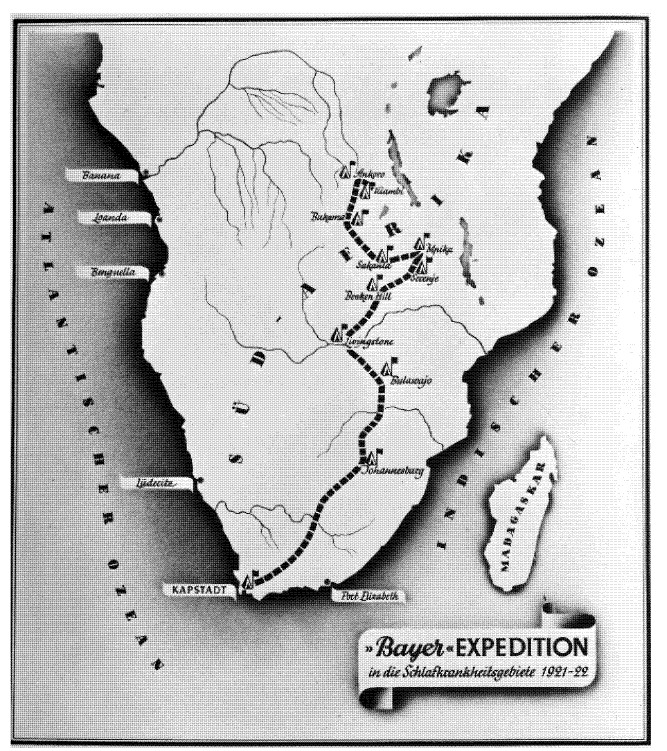
(Bayer Archive. 1921. (Picture 0-19682)) Route of the Bayer expedition starting on 2 November 1921 in Cape Town, travelling to the areas most affected by African sleeping sickness in what is today Tanzania, Burundi, Ruanda, DR Congo as well as a small part of Mozambique, Zimbabwe and Zambia, where the expedition ended in Kiambi in late 1922.

**Figure 2:**
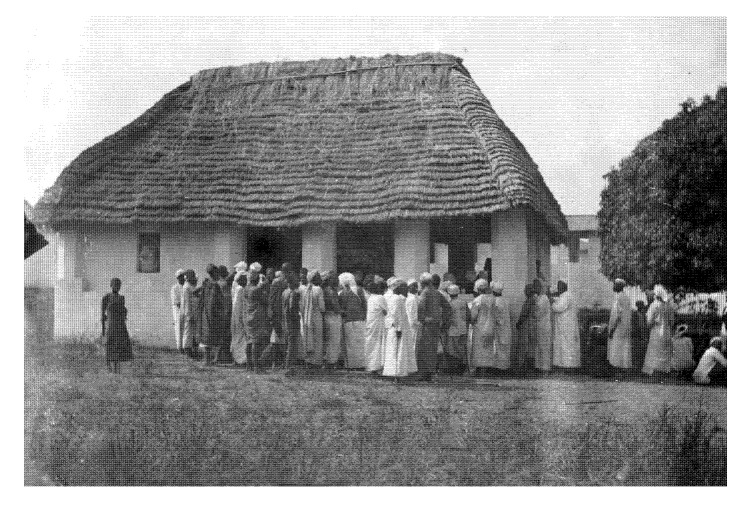
(Bayer Archive. 1921. (Picture 0-34295)) Screening of local population for symptoms of African sleeping sickness in the Urambi camp at Lake Tanganyika in 1921.

**Figure 3:**
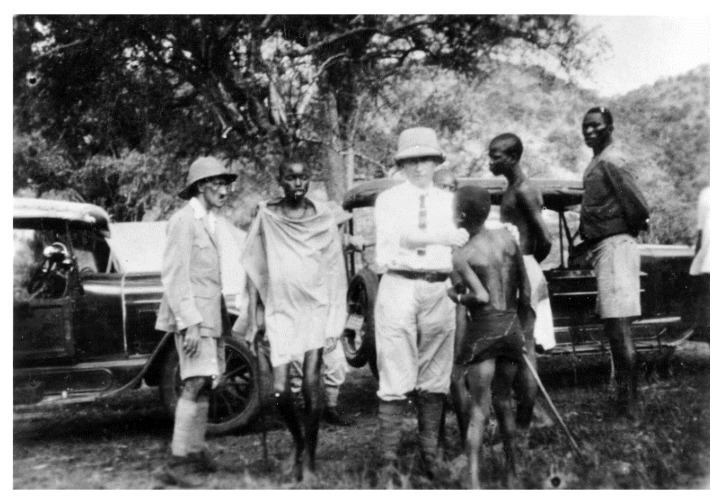
(Bayer Archive. Karl Friedrich Kleine with a Patient (Picture 0-3438301)) Friedrich Karl Kleine demonstrating the palpation of lymph nodes during screening of patients.

**Figure 4:**
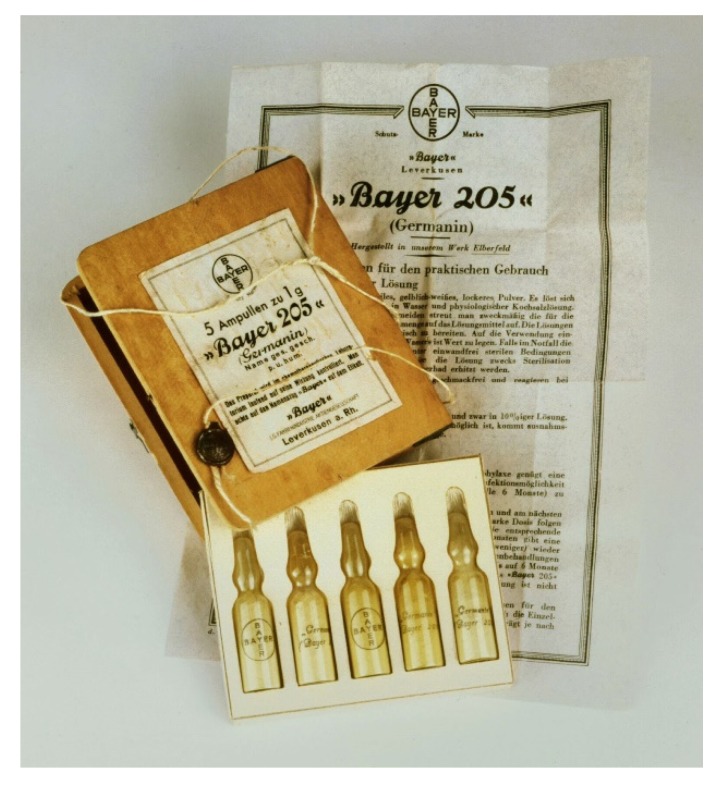
(Bayer Archive. Product “Bayer 205”, ca. 1934. (Picture 0-25791)) Pack of ampoules of “Bayer 205” (Germanin), ca. 1934.

**Figure 5:**
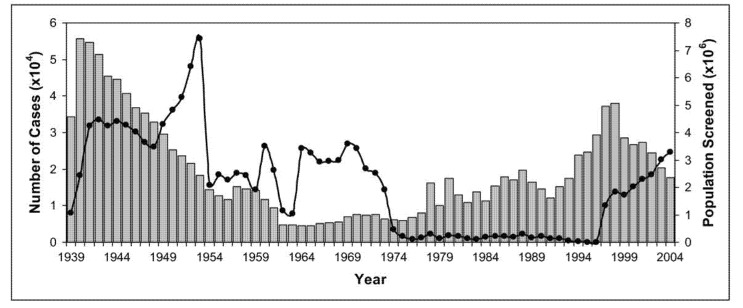
Number of cases of African sleeping sickness reported and population screened [5,10,11,12]. Graphs show the correlation between population screened (black circles) and number of reported cases (grey columns). Screening and surveillance of endemic disease areas are key to control African sleeping sickness.
